# A nano-preparation approach to enable the delivery of daphnoretin to potentiate the therapeutical efficacy in hepatocellular cancer

**DOI:** 10.3389/fphar.2022.965131

**Published:** 2022-09-28

**Authors:** Guanglin Zhu, Bing Wang, Guo Feng, Zhirong Zhou, Wei Li, Wen Liu, Hongmei Su, Wenjing Wang, Tiejie Wang, Xie-an Yu

**Affiliations:** ^1^ Department of Chinese Materia Medica, Guizhou University of Traditional Chinese Medicine, Guiyang, China; ^2^ NMPA Key Laboratory for Bioequivalence Research of Generic Drug Evaluation, Shenzhen Institute for Drug Control, Shenzhen, China

**Keywords:** daphnoretin, natural product, targeting delivery system for the liver, therapeutical efficacy in HCC, nano-preparation

## Abstract

Daphnoretin (DAP), isolated from a traditional Chinese medicine *Wikstroemia indica* (Linn. C. A. Meyer), could induce apoptosis of hepatocellular cancer (HCC) and inhibit tumor growth. However, the application of DAP in cancer therapies was hampered because to its poor solubility. Herein, this study aimed to design an approach of double-targeted nano-preparation to enable the delivery of DAP to potentiate the therapeutical efficacy in liver cancer via glycyrrhetinic acid-polyethylene glycol-block-poly (D,L-lactic acid)/polyethylene glycol-block-poly (D,L-lactic acid)-DAP (GPP/PP-DAP). In particular, the purity of separated DAP was up to 98.12% for preparation research. GPP/PP-DAP was successfully prepared by the thin-film hydration method. Subsequently, the GPP/PP-DAP was optimized by univariate analysis and the response surface methodology, producing a stable and systemically injectable nano-preparation. Impressively, on the one hand, cytotoxicity studies showed that the IC_50_ of the GPP/PP-DAP was lower than that of free DAP. On the other hand, the GPP/PP-DAP was more likely to be endocytosed by HepG2 cells and targeted to the liver with orthotopic tumors, potentiating the therapeutical efficacy in HCC. Collectively, both *in vitro* and *in vivo* results indicated the excellent tumor inhibition and liver targeting of GPP/PP-DAP, suggesting the nano-preparation could serve as a potential drug delivery system for natural ingredients with anti-hepatoma activity to lay the theoretical foundation for clinical application.

## Introduction

HCC is one of the most common malignancies with the third highest death rate in the world (Liu et al., 2022). An important feature of HCC is the poor prognosis; only about 20% of patients with early-stage liver cancer can be treated with surgery and liver transplantation (Anwanwan et al., 2020; Wu et al., 2020). Therefore, chemotherapy continues to play an important role in the treatment of liver cancer, including sorafenib, curcumin, and paclitaxel, but drug resistance and side effects are the major obstacles in treating patients with liver cancer (Cabibbo et al., 2021). Hence, it has great significance in developing new therapeutic agents for liver cancer. In recent years, multitudinous HCC therapy studies focused on natural products (Deng et al., 2022). *Wikstroemia indica* (Linn. C. A. Meyer), belonging to the *Wikstroemia* genus of the Thymelaeaceae family, was a traditional Chinese medicine, reporting with a variety of anti-tumor active compounds such as daphnoretin (DAP) (Shao et al., 2020; Shi et al., 2021). DAP, a dicoumarin derivative, could inhibit various cancer cell growth, especially inducing HCC apoptosis via an increase in Bax and p53 expression and a decrease in *Bcl2* expression (Yu et al., 2019; Badawy et al., 2021; Wang et al., 2021; Yao et al., 2021). However, DAP exhibited low water solubility and rapid clearance from the bloodstream, thus resulting in a short T1/2 in the plasma (3.5 h) (Hu et al., 2017; Liang et al., 2021). Therefore, it is imperative to develop a more effective therapeutic strategy to overcome the limitation of DAP in the treatment of HCC.

Nanotechnology was often used to overcome the limitations of active natural products. Specifically, nano-preparations provide an established approach to sustained release and increasing site-specific drug delivery by coupling the drug to targeted carrier materials, such as nano-micelles, nanocapsules, and spheres (Alyafee et al., 2017). Polyethylene glycol-block-poly (D,L-lactic acid) (PEG-PLA) was commonly used as the drug-loaded nanomaterial with biodegradable and biocompatible polymeric properties. The hydrophobic core of PEG-PLA could be used to encapsulate hydrophobic drugs, while the hydrophilic shell of PEG-PLA could effectively reduce the phagocytosis of the reticuloendothelial system, prolonging the circulation time of drug-loaded micelles *in vivo* (Deshantri et al., 2018). Accumulative studies had demonstrated that nanomaterials with diameters less than 200 nm could accumulate into solid tumors through the enhanced permeability and retention (EPR) effect (Spitzbarth et al., 2017). However, the EPR effect exhibited variability both between and within tumor types (Kalyane et al., 2019). In order to enhance the targeting effect, polymer materials were modified with special ligands. Through the interaction between the ligand and the receptor, the drug-loaded micelles were actively targeted to specific organs, tissues, or cells (Ryu et al., 2020; Zhang et al., 2020). Glycyrrhetinic acid (GA) was one of the main components of licorice (*Glycyrrhiza glabra* or *Glycyrrhiza uralensis*), which could target the liver cell membrane by specifically binding to the Glycyrrhetinic Acid Receptor (GA-R) (Sun et al., 2017; Sun et al., 2018). In addition, GA receptors also showed 1.5–5-fold higher expression in tumor tissue than in normal liver tissue (Zhang et al., 2018). Therefore, the specific binding of GA to its receptors enhanced the liver-targeting effects of drug delivery carriers, and GA-modified PEG-PLA probably was used as a nano-preparation approach for the delivery of natural products to the liver tumor site.

**SCHEME 1:**
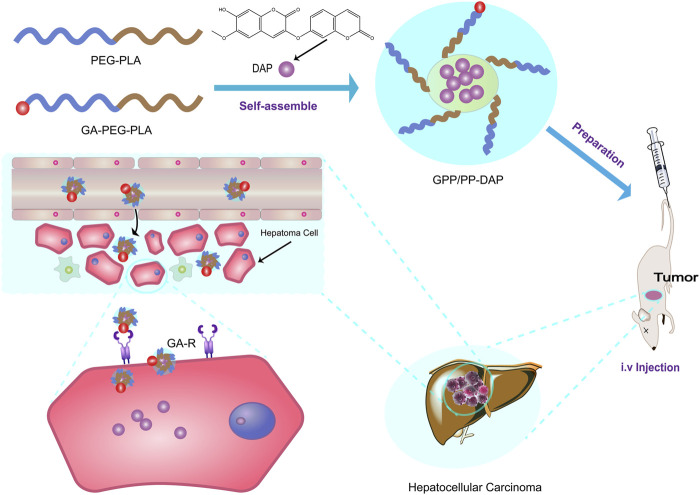
Schematic illustration of preparation and therapeutic process of GPP/PP-DAP.

In this study, a novel systemic nano-preparation of DAP-loaded GA-PEG-PLA/PEG-PLA (GPP/PP-DAP) as liver-targeting nanocarriers was successfully constructed (Scheme 1). To deliver sufficient DAP to target cells, the preparation conditions of GPP/PP-DAP were optimized by the univariate analysis and response surface methodology. Further, in evaluating the potential of this nano-preparation as an injection, the physicochemical properties of GPP/PP-DAP were characterized. As expected, the excellent liver-targeting and tumor inhibition properties of GPP/PP-DAP were verified at the cell and animal levels. Accordingly, this nanoparticle-mediated delivery platform could provide a broadly applicable strategy to effectively enhance the potency and liver-targeting ability of naturally active ingredients.

## Materials and methods

### Materials, cell culture, and animals

*Wikstroemia indica* were purchased from Guangxi Yulin Yinfeng International Chinese Medicine Port (Yulin, China; batch No. 20160115) and identified by Professor Wei Li, employed at Guizhou University of Traditional Chinese Medicine (Guiyang, China).

Standard daphnoretin (batch No. 111758-201101) was purchased from the China Institute of Food and Drug Verification (Beijing, China). PEG_2000_-PLA_2000_, PEG_2000_-PLA_5000_, PEG_3400_-PLA_2000_, and GA-PEG_3400_-PLA_2000_ were obtained from Xian Ruixi Biotechnology Co., Ltd. (Xian, China). DMSO, MTT, Triton X-100, and PBS buffer were purchased from Solarbio life Science (Shanghai, China). The Cell Counting Kit-8 (CCK-8) were obtained from Glpbio (Montclair, United States ).

Murine H22 and Human HepG2 hepatoma carcinoma cells were purchased from Zhongqiao Xinzhou Biotechnology (Shanghai, China). Cells were maintained in DMEM (Cellmax, China) supplemented with 10% (v/v) FBS (Solarbio, China) and 1% (v/v) penicillin–streptomycin (Gibco, United States ) at 37°C in 5% CO_2_.

Sprague–Dawley (SD) rats and Kunming (KM) mice were purchased from Tianqin Biotechnology Co. Ltd. (Changsha, China) and housed in standard rat cages at a temperature of 25°C with a humidity of 45%. All animals received humane care according to the Guide for the Care and Use of Laboratory Animals. Ethical approval (ethical clearance no.20210081) was obtained by the Institutional Animal Care and Use Committee of Guizhou University of Traditional Chinese Medicine.

### Separation and purity testing of DAP

Referring to the previous research results of our research group, the percolation method was used to prepare the ethanol extract of *Wikstroemia indica* (Feng et al., 2018). Then, the ethanol extract was separated by column chromatography on silica gel to obtain impure DAP. Finally, methanol reflux was used for purification.

The identification of DAP was performed on a hybrid quadrupole time-of-flight tandem mass spectrometry (X500R QTOF, AB SCIEX, Foster City, United States ) equipped with electrospray ionization (ESI) ion source. Data were analyzed using the AB SCIEX OS software integrated with the instrument. In addition, the ^1^H-nuclear magnetic resonance (NMR) and ^13^C-NMR dimethyl sulfoxide (DMSO-d6) spectra of the DAP were characterized at 500 Hz with an AVANCE III HD (500) NMR spectrometer (Bruker, Switzerland).

The purity of the sample was calculated according to the standard curve method. High-performance liquid chromatography (HPLC, Agilent Technologies, Waldbronn, Germany) with a C_18_ reverse-phase column (250 × 4.6 mm, 5 μm, Diamonsil, China) was used to detect the purity of DAP. The mobile phase consisted of methanol and deionized water (58:42, v/v). The flow rate was 1.0 ml/min, and the column temperature was maintained at 35°C. 10 µl sample injection was detected at 224 nm. DAP was injected into 36 μg/ml, 45 μg/ml, and 60 μg/ml solutions to calculate the purity according to the DAP standard.

### Preparation of PP-DAP and GPP/PP-DAP

**SCHEME 2:**
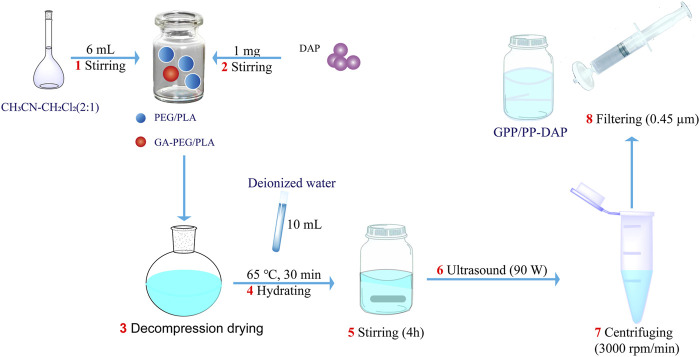
Schematic illustration of nano-preparation of GPP/PP-DAP.

DAP-loaded nanomicelles were prepared by the thin film hydration method (Scheme 2). First, PEG-PLA or GA-PEG-PLA/PEG-PLA was put into the vial, the organic solvent was added, and dissolved by stirring on the magnetic stirrer. Then DAP was added and stirred until completely dissolved. Second, the solution was transferred to a round-bottom flask, and the organic reagent was removed by a rotary evaporator at 45°C to obtain a blue polymer film. Then deionized water was added for hydration under atmospheric pressure. Third, the hydrated liquid was transferred to a vial, stirred for several hours at room temperature, sonicated for 6 min in the dark, and centrifuged at 3,000 rpm for 5 min. Finally, the nanomicelles were obtained by using a 0.45 μm micro-porous filter membrane.

### Optimizing the preparation of PP-DAP and GPP/PP-DAP

First, PEG_2000_-PLA_2000_, PEG_2000_-PLA_5000_, and PEG_3400_-PLA_2000_ were used to select carrier materials. The optimal drug loading materials were selected by comprehensive scores of entrapment efficiency (EE, accounting for 50%), drug loading (DL, accounting for 25%), and particle size (accounting for 25%) as evaluation indicators.

Second, the single factor experiment was used to identify the preliminary preparation conditions of PP-DAP. The ratio of DAP to PEG-PLA, organic solvent type and dosage, hydration temperature, hydration time and hydration water dosage, stirring time, and ultrasonic power of the preparation technology were successively investigated. The EE and DL were taken as an index to investigate the preliminary preparation technology.

Third, the Box–Behnken design was used to optimize the approach of nano-preparation. Based on the single factor result, the ratio of DAP to PEG-PLA, solvent volume, and hydration volume were the main factors influencing the EE and DL of PP-DAP. Therefore, using the same as the above comprehensive scores, the three factors mentioned above at three levels were used, and seventeen experiments were adopted according to the Box–Behnken design (Table 1). The three level-three variables were as follows: the ratio of drug to PEG-PLA (A; 5:1, 10:1, and 15:1, w/w); the solvent volume (B; 4, 5, and 6, v); and the hydration volume (C; 8, 10, and 12, v). Experimental data were analyzed by the Design-Expert program, and the three-dimensional response surfaces were built.

**TABLE 1:** Design and results of Box–Behnken experiment.

Test number	A: ratio of drug	B: solvent volume	C: hydration volume	EE (%)	DL (%)	Particle size (nm)	Score
1	10:1	5	10	73.05	9.74	136	0.66
2	15:1	5	12	70.92	6.30	117	0.60
3	10:1	6	8	71.22	9.60	125	0.69
4	10:1	4	12	67.42	8.77	118	0.64
5	10:1	6	12	71.23	9.63	115	0.75
6	5:1	4	10	49.16	11.35	132.1	0.38
7	10:1	5	10	74.66	9.91	136.2	0.69
8	15:1	5	8	72.87	5.95	137.4	0.50
9	5:1	5	8	47.55	10.52	157.4	0.18
10	15:1	6	10	80.21	7.10	113	0.80
11	5:1	5	12	52.27	12.30	155	0.34
12	10:1	4	8	74.30	9.83	153.2	0.59
13	10:1	5	10	76.47	10.16	133.4	0.74
14	5:1	6	10	50.73	11.40	142.5	0.35
15	10:1	5	10	75.86	10.05	125.2	0.78
16	15:1	4	10	78.00	6.82	128.5	0.66
17	10:1	5	10	74.30	9.89	115	0.80

Referring to the literature proportional feeding GA-PEG-PLA block copolymer (Zheng et al., 2019; Pan et al., 2020), one-ninth of GA-PEG_3400_-PLA_2000_ block copolymer was added to PEG_3400_-PLA_2000_ to prepare GPP/PP-DAP according to the result of the Box–Behnken design. Afterward, three samples of PP-DAP and GPP/PP-DAP were prepared in parallel to verify the process.

### EE and DL analysis

After careful preparation of DAP-loaded nanomaterials by the thin film hydration method, the EE% and DL% were determined by the HPLC method. Then, adding an equal volume of methanol, the solution was ultrasonically demulsified for 10 min. The result of the specificity study of DAP was shown in Supplementary Figure S1. Blank micelles did not affect its content determination. The EE and DL were calculated as follows:

$$EE\left(\%\right)=\frac{Encapsulated\;drug}{Total\;amount\;of\;DAP\;added}\times 100,$$

$$DL\left(\%\right)=\frac{Encapsulated\;drug}{nanomaterials\;weight}\times 100.$$

### Characterization and morphology evaluation of GPP/PP-DAP

To observe the size distribution and morphology of the PP-DAP and GPP/PP-DAP, the particle size distribution, Zeta potential, and polydispersity index (PDI) of PP-DAP and GPP/PP-DAP were measured by the Laser particle size analyzer (DMP3310, Beckman, United States ). Particle size data were expressed as the intensity-weighted distribution. Transmission electron microscopy (TEM) was performed using a transmission electron microscope (JEM-1400; JEOL, Tokyo, Japan) operating at an acceleration voltage of 120 kV. All experiments were performed in triplicate.

### *In vitro* stability, release, and hemolysis test of the nano-preparation

The micelle solutions were placed at 4°C and room temperature for 1 month, and experimental points were designed to measure the particle size distribution, Zeta potential, and PDI to evaluate the stability of micelles *in vitro*.

To evaluate the drug release kinetics *in vitro*, the dialysis method was utilized. DAP-loaded micelles (5 ml) were transferred to a dialysis bag (cut-off: 8–14 kDa). Then they were immersed in citrate buffer at pH 5.5 and in PBS buffer at pH 7.4 (20 ml, containing 0.5% Tween 80) with shaking for over 5 days at 37°C. At different time intervals, aliquots of 3.0 ml were withdrawn and immediately replaced with the same volume of fresh release media. DAP concentration was determined at 346 nm by the HPLC method.

According to previous reports (Steuber et al., 2016), hemolysis studies were carried out for PP-DAP and GPP/PP-DAP. The SD rat blood samples (8 weeks, 200–250g, abdominal aorta blood collection) were mixed with 0.5 times the normal saline and centrifuged at 3,600 rpm for 5 min. Then the supernatant was decanted, and the precipitate was rinsed three times. Equal-volume blood cells (2.5 ml, adjusted to a concentration of 2%) were mixed with saline solutions with different concentrations of DAP-loaded micelles, and the resulting suspensions were incubated at 37°C for 2 h. The samples were centrifuged at 3,600 rpm for 5 min. The absorbance of the supernatant was measured at 540 nm to reflect the amount of hemoglobin released. Zero and 100% hemolysis consisted of red blood cells suspended in physiological saline and Triton X-100 solution, respectively.

The percentage of hemolysis was calculated as per the following equation:

$$Hemolysis\;\left(\%\right)=\frac{{OD}_{X}-{OD}_{0}}{{OD}_{T}-{OD}_{0}}\times 100\;\left(\%\right),$$

where OD_X_ is the absorbance of the sample, OD_T_ is the absorbance of completely lysed red blood cells, and OD_0_ is the absorbance of zero hemolysis.

### Cellular uptake study

A fluorescence inverted microscope (CKX53; Majic, Shanghai, China) was used to compare the cellular uptake of free DAP, PP-DAP, or GPP/PP-DAP in HepG2 cells. HepG2 cells were seeded in 24-well plates (20,000 cells/well; 500 µl media) and incubated overnight. Meanwhile, HepG2 cells were treated with free DAP, PP-DAP, or GPP/PP-DAP (DAP concentrations: 1 μg/ml). After incubation for a certain period of time (1, 3, 6, 12, and 24 h), the cells were washed three times with PBS at 37°C. Fluorescence imaging was performed by using an inverted fluorescence microscope.

### *In vitro* cytotoxicity and biocompatibility evaluation study

First, the cell viability was measured by using the CCK-8 method. HepG2 cells were seeded in 96-well plates (5,000 cells/well; 100 µl media) and incubated overnight. Subsequently, the adherent cells were treated with free DAP, PP-DAP, and GPP/PP-DAP (DAP concentrations: 0.6, 25, 1.25, 2.5, 5, 10, 20, 40, and 80 μg/ml) for 48 h. Then, 10 µl CCK-8 solution was added to the medium, and the absorbance was measured at 650 nm after incubation for 2 h. The other cells were treated with GPP/PP-DAP for 24, 48, or 72 h to study the time dependence. Second, the MTT assay was used to investigate the biocompatibility evaluation of GPP/PP and PP micelles for L02 and HepG2 cells. The half maximal inhibitory concentration (IC_50_) was calculated by Graphpad prism 8 software.

### Establishment of an orthotopic mice tumor model

Five-week-old KM SPF mice (weighing 20 ± 2 g) were used for HCC implantation. The survival rate of cell suspension (H22, 2.5–3 × 107 in 1 ml physiological saline) was detected by Trypan blue staining. After the KM SPF mice were anesthetized, the skin and peritoneum were cut layer by layer along the liver to fully expose the left lobe of the liver. The cell suspension (20 µL H22) was pierced obliquely into the liver about 0.5 cm, then slowly injected into the liver. Finally, the abdominal cavity was sutured layer by layer. In order to determine the modeling time, mice were randomly dissected on the 3^rd^, 5^th^, 7^th^, and 10^th^ day to observe the growth of cancer cells.

### Evaluation of targeting ability *in vivo*

To evaluate *in vivo* tissue distribution and liver-targeting ability of DAP-loaded nano-preparations, the near-infrared fluorescence dye DiR iodide (DIR, meilunbio, China) was loaded and encapsulated into PP and GPP/PP according to the preparation process of drug-loaded nanomicelles.

The H22 orthotopic liver tumor model was established in KM mice according to the method described before. Each group of mice was intravenously injected with free DIR, PP-DIR, and GPP/PP-DIR at a dose of 5 mg/kg via tail vein and intraperitoneal injection. The mice biodistribution images of the nanoparticles were captured using the *in vivo* imaging system (IVIS) Spectrum (PerkinElmer, Santa Clara, CA, United States ) at different points. The mice were sacrificed at 48 h, and the major organs, including the heart, liver, spleen, kidneys, lung, and tumors, were collected for *ex vivo* tissue fluorescence imaging.

### *In vivo* anti-tumor efficacy

The mice were randomly divided into six groups (6 mice in each group). The three groups of orthotopic liver tumor mice were administered free DAP, PP-DAP, and GPP/PP-DAP (21 mg/(kg/d)), the positive control group was administered cyclophosphamide (CTX, Baxter, United States; 15 mg/(kg/d)), and the blank and model control groups were given the same volume of normal saline by intraperitoneal injection. After continuous administration for 7 days, the blood sample was taken 24 h after the last administration. The contents of AST, ALT, TNF-α, and IL-2 in serum were assayed by an ELISA kit. The ELISA assay was conducted according to the handbook provided by the ELISA kit (Jingmei, Jiangsu, China). The organ index of each group was calculated after the mice were dissected. The liver tissues were paraffin-embedded, dewaxed, rehydrated, and stained with HE, and the images were captured by an inverted Olympus BX53 microscope (Olympus, Tokyo, Japan).

### Statistical analysis

SPSS 26.0 software was used to analyze the data. The results were presented as the means ± SD. *p < 0.05 was regarded as significant, while **p < 0.01 was regarded as highly significant.

## Results

### Preparation and purity testing of DAP

**FIGURE 1:**
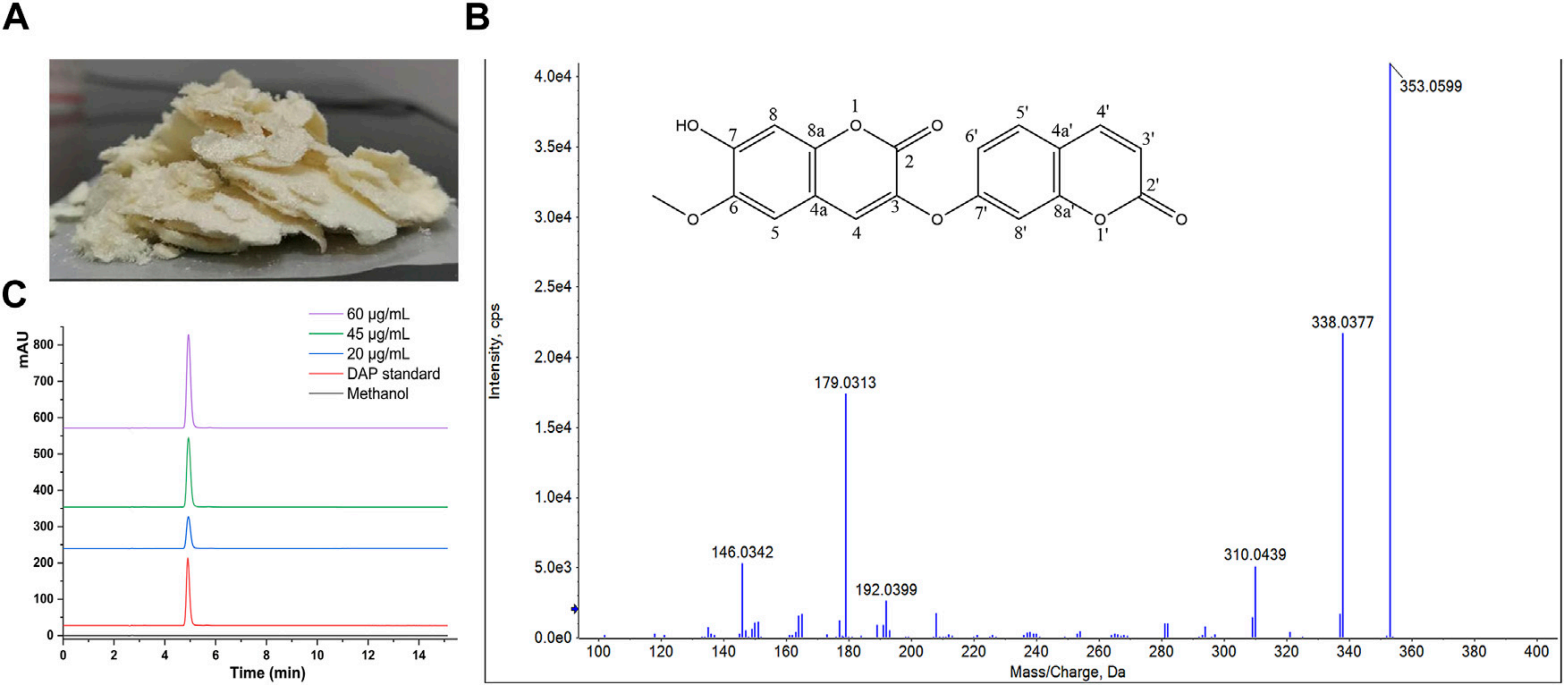
**(A)** Sample of DAP. **(B)** Structural formula and mass spectrum of DAP. **(C)** HPLC chromatogram of 20, 45, and 60 μg/ml DAP sample for purity testing.

The yield of dry extract was 10.08% from 5 kg powders of *Wikstroemia indica*. At the last eluted, yellowish floc was precipitated. After purification, 4.5964 g yellowish compound was seen in Figure 1A. The yield of DAP was 0.92 g/kg. The compound had major ions at m/z 353.0599 [M + H]^+^ (Figure 1B) and the formula was calculated as C_19_H_12_O_7_. ^1^H-NMR and ^13^C-NMR spectrum of the DAP sample were shown in Supplementary Figure S2. ^1^H-NMR (500 MHz, DMSO-d_6_) δ: 3.81 (s, 3H, OCH3), 6.36 (d, 1H, J = 9.6 Hz, H-3′), 6.86 (d, 1H, J = 2.4 Hz, H-8′), 7.11 (dd, 1H, J = 8.8, 2.4 Hz, H-6′), 7.12 (s, 1H, H-8′), 7.20 (s, 1H, H-5), 7.70 (d, 1H, J = 8.4 Hz, H-5′), 7.86 (s, 1H, H-4), 8.02 (d, 1H, J = 9.2 Hz, H-4′). ^13^C-NMR (126 MHz, DM SO-d_6_) δ: 156.87 (C-2), 135.62 (C-3), 130.77 (C-4), 110.08 (C-4a), 109.29 (C-5), 145.56(C-6), 150.25(C-7), 102.67(C-8), 147.32 (C-8a), 159.87 (C-2′), 114.0 (C-3′), 144.1 (C-4′), 114.5(C-4’a), 130.0 (C-5′), 113.5(C-6′), 159.87 (C-7′), 103.91 (C-8′), 154.90 (C-8’a), 55.92 (6-OCH3). These aforementioned data were consistent with the reference (Shi et al., 2021), which confirmed that this compound was DAP. Besides, the DAP standard was used to draw the standard curve (y = 35.67x + 25.499, *R* ^2^ = 0.9998, linear range, 0.5∼80 μg/ml). The method validations of DAP were given in Table 2. For the repeatability, precision, and stability testing, the *RSD* values of the average absorbance were all less than 2%. The mean recovery values of DAP ranged from 97.00–99.20%. The purity of the three samples was calculated to be 98.12%, indicating that the self-made DAP sample could be used in further preparation study (Figure 1C).

**TABLE 2:** Method validations of DAP by HPLC.

Precision (%)			Repeatability (%)	Stability (%)	Recovery (mean ± SD)		
2.5	10	40			5	10	20
0.87	0.38	0.46	1.81	1.71	97.53 ± 0.5	99.00 ± 0.17	98.93 ± 0.24

### Optimizing the preparation of GPP/PP-DAP

In order to improve the water solubility and bioavailability of DAP, the DAP-loaded amphiphilic nano-preparation (PP-DAP and GPP/PP-DAP) was prepared. First, it was found that the EE and DL of PEG_3400_-PLA_2000_ DAP-loaded nanomicelles were higher, and the particle size was smaller and more uniform (Supplementary Figure S3). Therefore, PEG_3400_-PLA_2000_ was chosen as the micellar carrier material. Next, a single factor way was used to optimize the preparation technology of nano-preparation. According to the results of the single factor way (Supplementary Figure S4), a preliminary parameter of preparation technology was selected: the ratio of DAP to PEG-PLA was 10:1; 5 ml acetonitrile-dichloromethane (2:1) was selected to dissolve DAP and nanomaterials; deionized water (10 ml) was used to hydrate 30 min at 65°C; the stirring time was 4 h; and the ultrasonic power of the probe was set to 90 w.

Exploring it further, the results of the Box–Behnken design used Design Expert 8.0.6 software to calculate the comprehensive score, which was used as the response value. The regression equation was as follows: Y = 0.73 + 0.16A + 0.04B + 0.046C + 0.042AB-0.015AC+0.0025BC-0.22A^2^ + 0.038B^2^-0.1C^2^ (*R* ^2^ = 0.9618). The results were shown in Table 3. The *p*-value of the regression equation was significantly smaller than 0.01, which indicated that this method was reliable. In addition, the *p*-value of lack of fit was significantly larger than 0.05. It indicated that the reasons for lack of fit did not exist, and the fitted regression equation was feasible. These data presented that this model can analyze the preparation process.

**TABLE 3:** ANOVA for the response surface quadratic model.

Test number	A: ratio of drug	B: solvent volume	C: hydration volume	EE (%)	DL (%)
Model	0.52	9	0.058	19.58	0.0004
A-A	0.21	1	0.21	72.21	< 0.0001
B-B	0.013	1	0.013	4.31	0.0766
C-C	0.017	1	0.017	5.76	0.0475
AB	7.225 × 10^−3^	1	7.225 × 10^−3^	2.43	0.1628
AC	9 × 10^−4^	1	9 × 10^−4^	0.3	0.5992
BC	2.5 × 10^−5^	1	2.5 × 10^−5^	8.415 × 10^−3^	0.9295
A2	0.21	1	0.21	71.43	< 0.0001
B2	6.08 × 10^−3^	1	6.08 × 10^−3^	2.05	0.1956
C2	0.046	1	0.046	15.48	0.0056
Residual	0.021	7	2.971 × 10^−3^	-	-
Lack of fit	6.875 × 10^−3^	3	2.292 × 10^−3^	0.66	0.619
Pure error	0.014	4	3.48 × 10^−3^	-	-
Cor total	0.54	16	-	-	-

**FIGURE 2:**
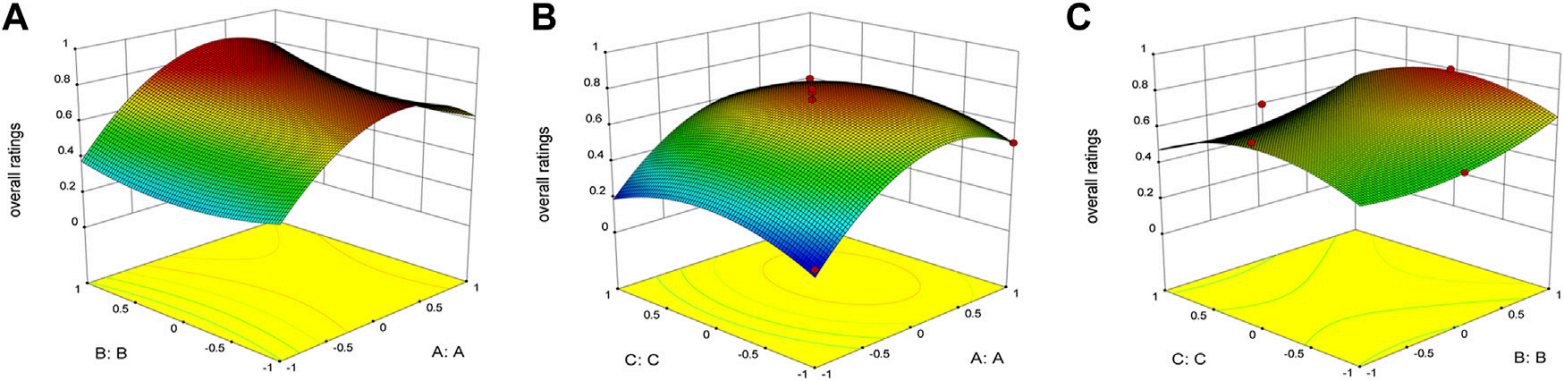
3-D response surface plots indicating the influence of organic solvent volume **(A)**, hydration volume **(B)**, and PEG-PLA to DAP ratio **(C)** on the encapsulation rate, drug loading, and particle size.

The three-dimensional (3-D) surface images between the dependent and independent variables were showen in Figure 2. Three response surfaces were all downward convex surfaces, and the center in the contour diagram lay in the scope of three levels, which indicated that the maximum response value existed in the range of three levels (Pasandide et al., 2018). The maximum entrapment rate of response value presumed by the regression model was 0.80. The influence of the three factors on the comprehensive score was sorted: the ratio of drug to PEG-PLA > hydration volume > solvent volume. After software analysis, the optimal preparation technology parameters of DAP-loaded nanomicelles were obtained: the drug-loading ratio was 15:1, the amount of organic solvent was 6 ml, and the hydration volume was 10 ml. The EE value of PP-DAP and GPP/PP-DAP nano-preparation were 76.04 ± 0.80% and 72.27 ± 1.29%, and the DL value were 11.13 ± 0.28% and 8.89 ± 0.28%, respectively. The actual response value was 0.82, which had a little deviation from the theoretical score. It all showed that GPP/PP nano-preparation was feasible to encapsulate the water-insoluble DAP.

### Characterization of the DAP-loaded nano-preparation

**FIGURE 3:**
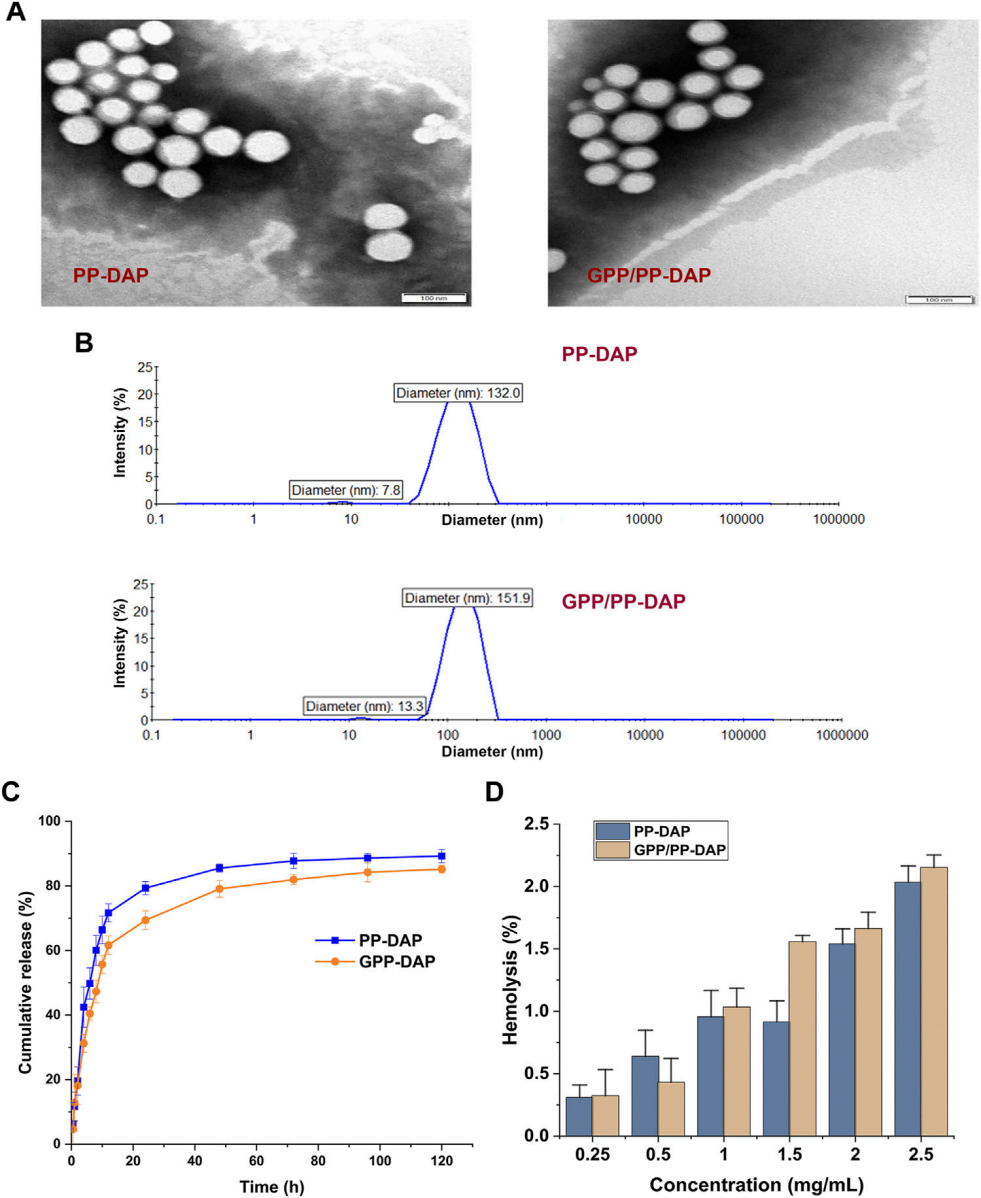
**(A)** TEM image of nanomicelles, bar: 100 nm. **(B)** Particle size distribution of the DAP-loaded nano-preparation. **(C)** Cumulative release curve of PP-DAP and GPP/PP-DAP in the release medium at pH 5.5. **(D)** Hemolysis rate analysis of PP-DAP and GPP/PP-DAP.

TEM images revealed that the size of PP-DAP and GPP/PP-DAP were less than 100 nm with a roughly spherical structure (Figure 3A). In addition, the particle size of PP-DAP was around 132.0 nm with PDI = 0.196 and Zeta potential of −11.98 ± 0.74 mV, and GPP/PP-DAP was around 151.9 nm with PDI = 0.173 and Zeta potential of −13.82 ± 0.66 mV (Figure 3B). As of the 28th day, there was no significant difference in the particle size and Zeta potential changes of PP-DAP and GPP/PP-DAP nano-preparation at 25 and 4°C compared with the first day 0 (*p* > 0.05) (Supplementary Figure S5). In general, two nano-preparations were stable within 28 days, which could minimize the risk of capillary embolism (Ghezzi et al., 2021). As shown in Figure 3C, the cumulative release rate of the two DAP-loaded nano-preparations could achieve 80% in a weak acid environment. Hence, they could rapidly release DAP within 24 h.

Actual photos of the hemolysis experiment of PP-DAP and GPP/PP-DAP were shown in Supplementary Figure S6. After calculating, hemolysis of nano-preparations was positively correlated with the concentration (Figure 3D). When the micelle concentration was within 2 mg/ml, the hemolytic activity of PP-DAP and GPP/PP-DAP nano-preparations with respect to RBCs was negligible. Therefore, it came to the conclusion that GPP/PP-DAP nano-preparation was biocompatible for drug delivery of intravenous administration.

### Evaluation *in vitro*

**FIGURE 4:**
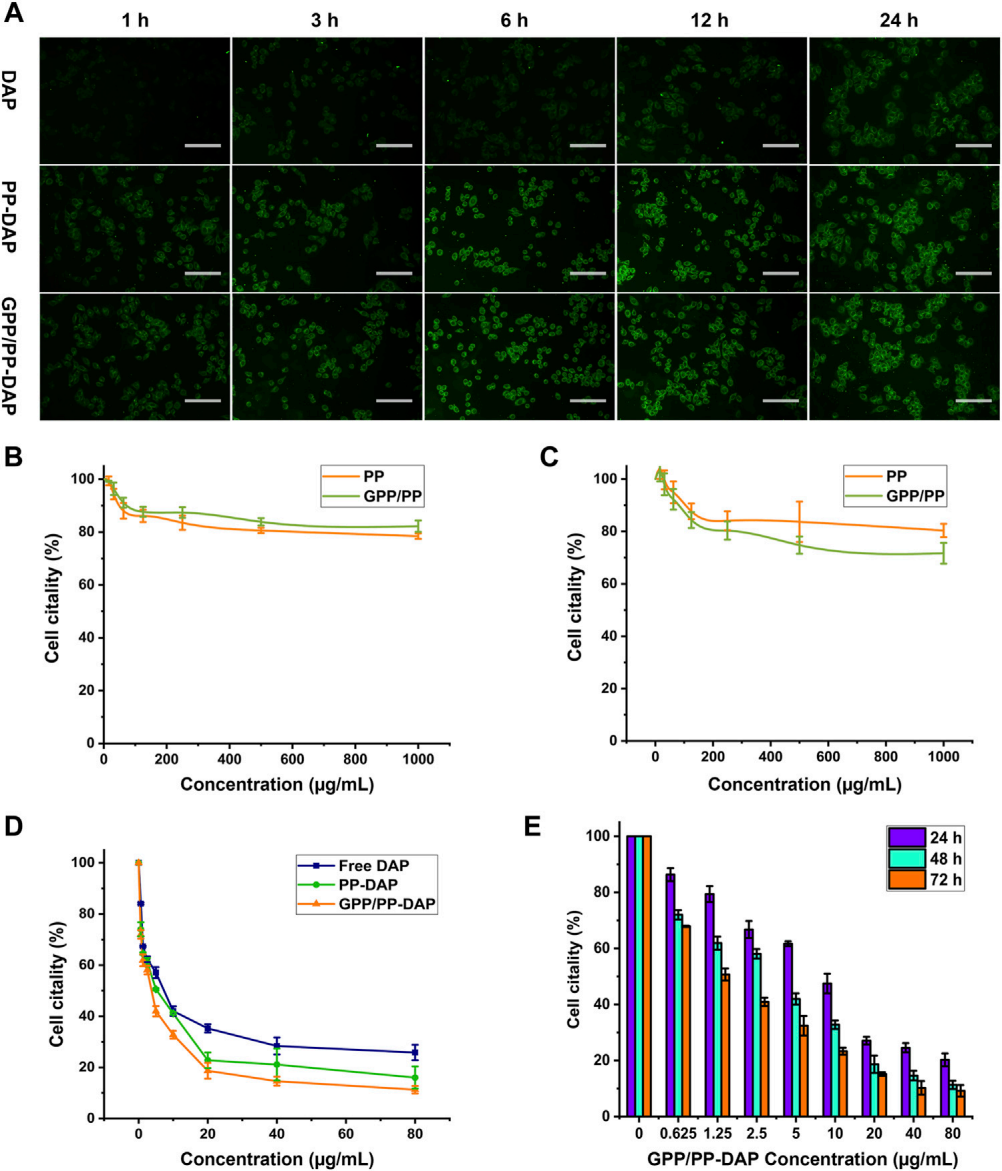
**(A)** Fluorescence images of HepG2 cells incubated with DAP, PP-DAP, or GPP/PP-DAP foe different times at the concentration of DAP equivalent: 1 μg/ml; scale bar: 50 μm. Biocompatibility of blank micelles to HepG2 cells **(B)** and L02 cells **(C)**. **(D)** Cytotoxicity of DAP, PP-DAP, and GPP/PP-DAP to HepG2 cells after 48 h treatment. **(E)** Cytotoxicity of GP/PP-DAP to HepG2 cells at 24, 48, or 72 h.

As a coumarin derivative with fluorescent, the Ex/Em wavelength of DAP was 380/442 nm (Supplementary Figure S7). Cellular uptake of DAP-loaded nanomicelles was evaluated by targeting *in vitro*. The results of the uptake of DAP-loaded nanomicelles by HepG2 cells were shown in Figure 4A. HepG2 cells take up DAP in a time-dependent manner. The fluorescence signals of the preparation groups were significantly stronger than the free DAP group at all time points. The fluorescence signals of the GPP/PP-DAP group were slightly stronger than the PP-DAP group. These data indicated that the enhanced uptake and absorption of DAP by cells were due to the self-assembly of nanomicelles and GA ligands.

The two blank micelles had almost no effect on inhibiting the proliferation of L02 and HepG2 cells. Although the concentration of blank micelles is increased to 1 mg/ml, the survival rate of L02 and HepG2 cells can still reach 80%, which confirmed that blank micelles had lower cytotoxicity than L02 and HepG2 cells (Figures 4B,C). Moreover, the IC_50_ of free DAP, PP-DAP, and GPP/PP-DAP were 19.13, 9.90, and 9.48 μg/ml, respectively (Figure 4D). In addition, GPP/PP-DAP nano-preparation inhibited the viability of HepG2 cells in a dose-dependent and time-dependent manner (Figure 4E). These studies had shown that the preparation of the DAP into nanomaterials could improve the inhibitory effect on HepG2 cells.

### Evaluation of the targeting *in vivo* on orthotopic liver tumors in mice

In order to intuitively study the target and efficiency *in vivo*, the orthotopic liver tumor model was used to evaluate the anti-tumor effect of nano-preparations. After modeling, the mouse liver anatomies were shown in Supplementary Figure S8. On the seventh day, the liver became pale with spotty solid tumors on the surface. On the tenth day, the liver had obvious lesions, and the volume of solid tumors increased significantly. In the late stage of cancer, drugs could not effectively improve the deterioration of the disease (Hong et al., 2020). In order to effectively exert the effect, this experiment determined the modeling days as 7 days.

**FIGURE 5:**
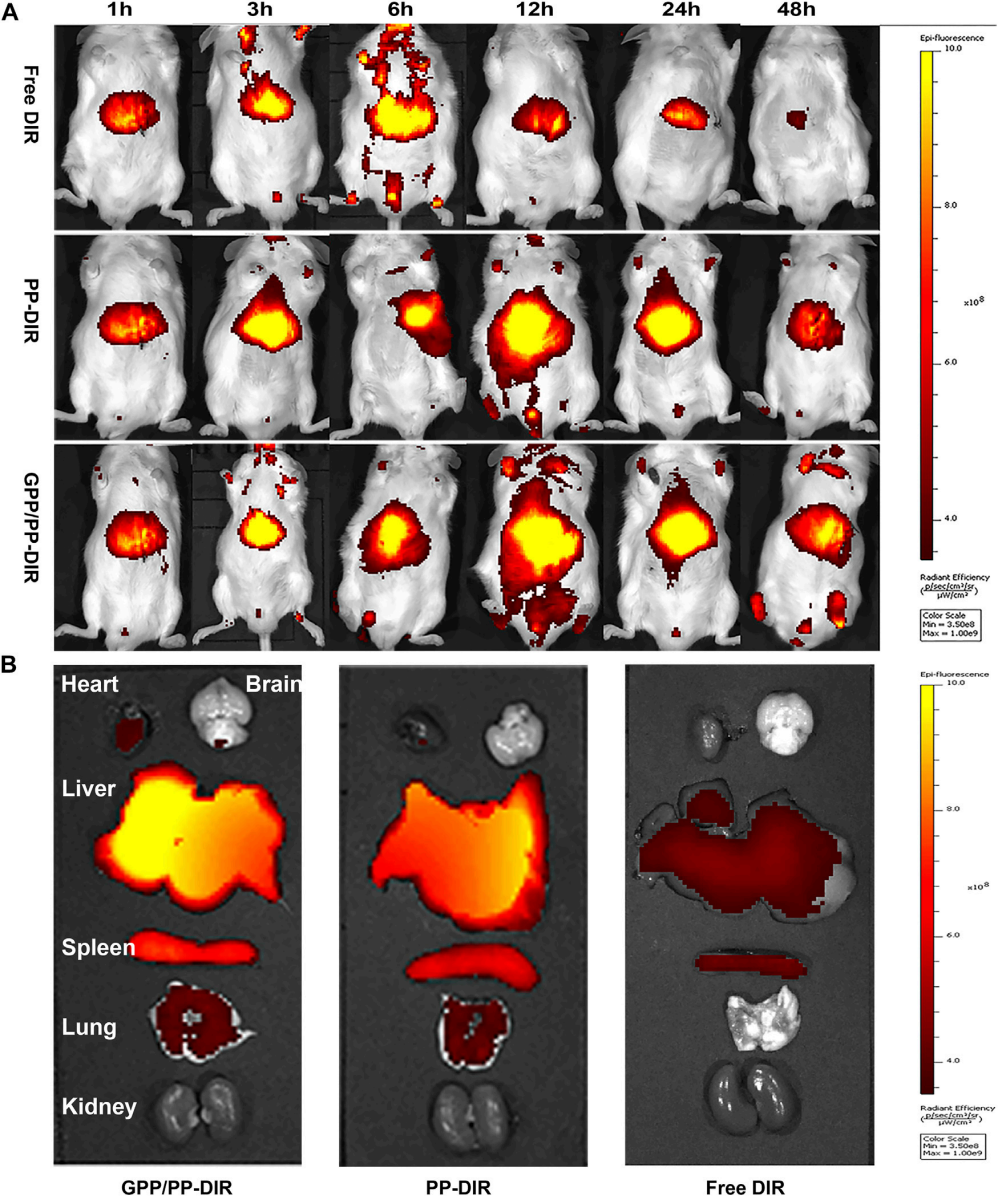
**(A)** Fluorescence *in vivo* imaging of mice injected with DIR, PP-DIR, and GPP/PP-DIR for different times. **(B)** The fluorescence distribution of mice viscera at 48 h. These mice were administered different DIR-tagged formulations via tail vein injection at a dose of 0.5 mg/kg.

*In vivo* imaging study and tumor distribution, free DIR and DIR-loaded nano-preparation were immediately distributed to the liver region after caudal vein injection. Free DIR fluorescence was the strongest at 6 h and almost disappeared at 48 h. In nano-preparation groups, the fluorescence was the strongest at 12 h. The liver-targeting efficiency of the GPP/PP-DIR group was 3.23-fold higher than that in the Free DIR group at 24 h. In addition, there was still fluorescence distribution in the liver at 48 h, and the fluorescence in GPP/PP-DIR group was stronger than that in the PP-DIR group (Figure 5A). Further information was provided by the *ex vivo* fluorescence imaging of major organs and tumors after 48 h injection, which showed similar results as the *in vivo* imaging (Figure 5B). The results of the intraperitoneal injection again verified that GA-modified PEG-PLA increased drug accumulation to a greater extent at time points (Supplementary Figure S9). Hence, the tumor-targeting ability allows our nano-preparation to effectively accumulate in tumor sites, which was the premise to obtain better efficacy.

### Assessment of the anti-tumor efficacy of GPP/PP-DAP

**FIGURE 6:**
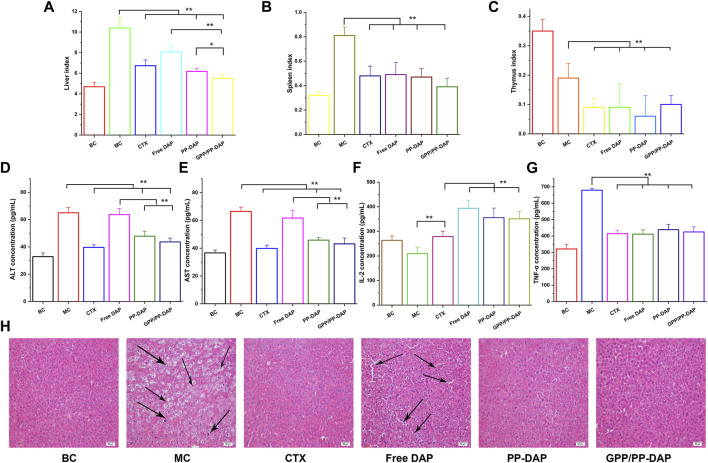
**(A–C)** Changes of liver and immune organ index in each group (mg/g). **(D–E)** Plasma ALT and AST levels of mice. **(F–G)** The levels of TNF-α and IL-2 in mice plasma. The values are presented as mean ± SD (*n* = 6), **p < 0.01, *p < 0.05. **(H)** Histopathological observation of tumor in different groups (HE staining; magnification, ×200). Scale bars: 50 µm.

We next evaluated whether nano-preparations could suppress HCC growth using the orthotopic liver tumors model (Figure 6A). The liver index of the different groups revealed that the liver index of the normal saline groups rapidly increased, reaching over 2.21-fold bigger than the blank group. Moreover, GPP/PP-DAP treatment significantly induced apoptosis in tumor cells compared with the free DAP group (*p* < 0.01) and PP-DAP group (*p* < 0.05). Similarly, the spleen and thymus index of nano-preparations groups were significantly changed compared with that of the model group (Figures 6B,C), which reflected that nano-preparations could regulate immune function (Sun et al., 2019).

The hepatic injury of each group was illustrated in Figures 6D,E. Serum ALT and AST concentrations of GPP-DAP, PP/PP-DAP, and CTX groups were significantly lower than those in the model group (*p* < 0.01). Furthermore, the ALT and AST concentrations in GPP/PP-DAP groups were significantly lower than those in the free DAP group (*p* < 0.01). The changes in IL-2 concentrations were illustrated in Figure 6F. The concentration of IL-2 in the treatment group was significantly increased compared with that in the model group (*p* < 0.01). Meanwhile, the groups containing DAP were significantly higher than the CTX group (*p* < 0.01). As the multifunctional cytokine with direct anti-tumor effects, the TNF-α contents in the treatment group were significantly reduced compared with the model group in Figure 6G (*p* < 0.01). Besides, the concentration of TNF-α in GPP/PP-DAP groups was significantly lower than that in the free DAP group (*p* < 0.01).

To further investigate the anti-tumor effect of nano-preparations, we used HE staining for further verification. As shown in Figure 6H, the tumor cells of the model group were heteromorphic, the volume of tumor cells increased, and the ratio of nucleoplasm increased. The liver tissue of the free DAP group observed the infiltration of inflammatory cells, the ratio of nucleoplasm increased, and showed obvious gaps. However, liver cells of the normal saline and GPP/PP-DAP groups exhibited no significant damage. Generally, it was suggested that DAP-loaded nano-preparations could significantly inhibit hepatoma growth, especially in the GPP/PP-DAP groups.

## Discussion

At present, research on the anti-tumor activity of natural products had become a hot topic. However, the inefficient delivery of these drugs to the liver tumor site due to complex *in vivo* environments also limited their application (Wang et al., 2016). A good anti-tumor effect of DAP had been demonstrated, particularly for HCC (Yu et al., 2019; Badawy et al., 2021). Nevertheless, the pharmacokinetic study of DAP reported that the pharmacokinetic parameters Tmax, Cmax, AUC0-t, T_1/2_, and MRT were 2.92 h, 178.00 μg/L, 905.89 μg/L•h, 3.50 and 6.95 h, respectively (Hu et. Al., 2017). As a potential therapeutic agent for liver cancer, the low water solubility and rapid removal from the bloodstream still limited the application of DAP.

Nanotechnology provided possibilities for enhancing drug targeting, prolonging drug action time, and reducing drug toxicity and dosage (Spitzbarth et al., 2017). Hepatic-targeted DAP-loaded delivery systems may help to overcome the adverse effects associated with chemotherapy for HCC. In this study, we described the synthesis of GA-modified PEG-PLA and PEG-PLA nanoparticles encapsulating DAP using the thin film hydration method. Its hydrophobic core could be used to encapsulate DAP, and the hydrophilic shell could effectively reduce the phagocytosis of the reticuloendothelial system, prolonging the circulation time (Spitzbarth et al., 2017). Moreover, this nano-preparation could deliver the drug to the liver tumor site by EPR effect and liver targeted GA.

In this experiment, a single factor way and the Box–Behnken design were combined to optimize the preparation technology of nano-preparation. The EE and DL values of GPP/PP-DAP nano-preparation were 72.27 ± 1.29% and 8.89 ± 0.28%, respectively. The actual response value was not different from the theoretical value, which indicated that the optimized preparation technology was stable and reliable (Pasandide et al., 2018).

The size of the nano-preparation < 200 nm had more uptake and retention in the tumor due to the promotion of the EPR effect in the tumor tissue (Ghezzi et al., 2021). In this study, the Zeta potential of the GPP/PP-DAP was −13.82 ± 0.66 mV. When the Zeta potential of the micelle particles was negative, it could reduce the agglutination of blood cells and the risk of hemolysis via intravenous injection (Deshantri et al., 2018). PDI value of 0.173 relatively monodispersed particle sizes. Moreover, nano-preparation was relatively stable within 1 month to minimize the risk of capillary embolism (Ryu et al., 2020). Controlled and continuous drug release by drug carriers can improve drug bioavailability and reduce the side effect and toxicity in normal tissues (Wang et al., 2016). The release of PP-DAP and GPP/PP-DAP were all enhanced up to 80%. The pattern of rapidly releasing in the weak acid environment indicated the possibility for the targeted treatment of DAP (Alyafee et al., 2017). The hemolytic activity with biocompatible was less than 2%, confirming the provisions of injectable preparation (Steuber et al., 2016). Thus, the obtained results of characterization indicated the DAP-loaded nano-preparation candidates for precise therapy.

Next, we evaluated the liver-targeting effect and anti-tumor efficiency of DAP-loaded nano-preparations *in vitro*. A cellular uptake study showed that the fluorescence signal of the GPP/PP-DAP group was significantly stronger compared with that of the free DAP group. In addition, GPP/PP-DAP (IC_50_: 9.48 μg/ml) also showed excellent cytotoxicity compared with free DAP (IC_50_: 19.13 μg/ml). Some studies verified that GA-modified nanomaterials could make the formulation solution more compatible with liver tumor cells (Sun et al., 2017; Chang et al., 2018). Therefore, we speculated that DAP-loaded nano-preparation with better cytotoxicity could take more DAP in HepG2 cells by liver targeting the affected tissues and prolonging the time of drug action.

To evaluate anti-tumor efficiency and liver-targeting ability *in vivo*, an orthotopic liver tumor model was established using the H22 cells, which was an ideal animal model for basic research of liver cancer (Streubel et al., 2021). Furthermore, real-time images *in vivo* and tissue distribution showed that GPP/PP nanomicelles increased DiR accumulation in liver tissues compared with the free DiR and PP-DiR groups, indicating that GPP/PP nanomicelles could enhance the targeted delivery of DAP to the liver. Preliminary experiments observed that a small number of cancer cells spread to the abdominal cavity, forming liver cancer in mice with ascites cancer cells. Furthermore, considering that the drug was administered intraperitoneally, an intraperitoneal injection was designed in this study to verify its targeting. As shown in Figure 5 and Supplementary Figure S8, both drug delivery strategies of the GPP/PP-DAP group showed good liver targeting.

In our studies, GPP/PP-DAP treatment significantly inhibited orthotopic liver tumor growth compared with free DAP and PP-DAP groups. Together, the ALT and AST concentrations in GPP/PP-DAP nano-preparation were significantly lower compared with free DAP (*p* < 0.01). The ALT and AST levels are usually used to evaluate hepatic injury (Mu et al., 2020). Due to the state of suspension, a lot of free DAP in the abdominal cavity could not be absorbed in time, which increased its burden without reversing liver injury. In addition, the serum level of interleukin-2 (IL-2) in the clinical was used as the indicator for disease observation and drug efficacy (Balkwill, 2006; Kolios et al., 2021). All DAP groups significantly promoted IL-2 secretion compared with the CTX group (*p* < 0.01). Hence, we speculated that the anti-tumor effect of DAP probably is related to the promotion of IL-2 secretion. The interaction between programmed cell death protein (PD-1) and programmed cell death ligand (PD-L1) inhibited T cell activity and the release of IFN-γ and IL-2, which produced a temporary immunosuppressive signal to reduce anti-tumor response ability (Gao et al., 2009). Triple immunotherapy of anti-PD-1, anti-PD-L1, and sorafenib reduced the metastasis and growth of HCC (Siu et al., 2017). Further, IL-2 augments the sorafenib-induced apoptosis in liver cancer by promoting mitochondrial fission and activating the JNK/TAZ pathway (Ding et al., 2018).

## Conclusion

To sum up, the anti-hepatoma active ingredient (DAP) extracted from a traditional Chinese medicine was successfully co-loaded in a nanomaterial-mediated delivery platform, which can be used as a novel double-targeting nano-preparation for the delivery of DAP into the liver effectively to potentiate the therapeutical efficacy in HCC. Accordingly, establishing a novel liver-targeting delivery platform not only showed that the constructed GPP/PP-DAP contained the properties of active and passive hepatoma targeting by integrating PEG-PLA with GA but also provided a new strategy for effective therapy and the clinical transformation of these naturally active components.

## Data Availability

The original contributions presented in the study are included in the article/Supplementary Material; further inquiries can be directed to the corresponding authors.

## Abbreviations

CTX, cyclophosphamide; DAP, daphnoretin; DiR, 1,1ʹ-dioctadecyl-3, 3, 3ʹ, 3ʹ-tetramethyl indotricarbocyanine iodide; DL, drug loading; DMSO, dimethyl sulfoxide; EE, entrapment efficiency; EPR, enhanced permeability and retention; ESI, electrospray ionization; GA, glycyrrhetinic acid; GA-R, glycyrrhetinic acid receptor; GPP/PP-DAP, glycyrrhetinic acid-polyethylene glycol-block-poly (D,L-lactic acid)/polyethylene glycol-block-poly(D,L-lactic acid)-DAP; HCC, hepatocellular cancer; HPLC, high-performance liquid chromatography; IVIS, *in vivo* imaging system; PDI, polydispersity index; PEG-PLA, polyethylene glycol-block-poly (D,L-lactic acid); NMR, nuclear magnetic resonance; TEM, transmission electron microscopy.
