# Supplementary material for: A nano-preparation approach to enable the delivery of daphnoretin to potentiate the therapeutical efficacy in hepatocellular cancer
Source: Front Pharmacol. 2022 Sep 28;13:965131. doi: 10.3389/fphar.2022.965131 (PMC9554561; doi:10.3389/fphar.2022.965131)

**Supporting Informations**

**A Nano-preparation Approach Enable Delivery of Daphnoretin to Potentiate the Therapeutical Efficacy in Hepatocellular Cancer**

Guanglin Zhu ^a,1^, Bing Wang ^b,1^, Guo Feng ^a,^*, Zhirong Zhou ^a^, Wei Li ^a^, Wen Liu ^a^, Hongmei Su ^a^, Wenjing Wang ^a^, Tiejie Wang ^b^, Xie-an Yu ^b,^*

^a^Department of Chinese Materia Medica, Guizhou University of Traditional Chinese Medicine, Guiyang, Guizhou 550025, P.R. China

^b^NMPA Key Laboratory for Bioequivalence Research of Generic Drug Evaluation, Shenzhen Institute for Drug Control, Shenzhen 518057, P.R. China

^1^These authors contributed equally.

*Corresponding author: Guo Feng: [453989352@qq.com;](mailto:453989352@qq.com;) Xie-an Yu: yuxieanalj@126.com

# Figure S1. The HPLC specificity study of DAP.





# Figure S2. The ^1^H-NMR (A) and ^13^C-NMR (B) spectrums of DAP samples.

**
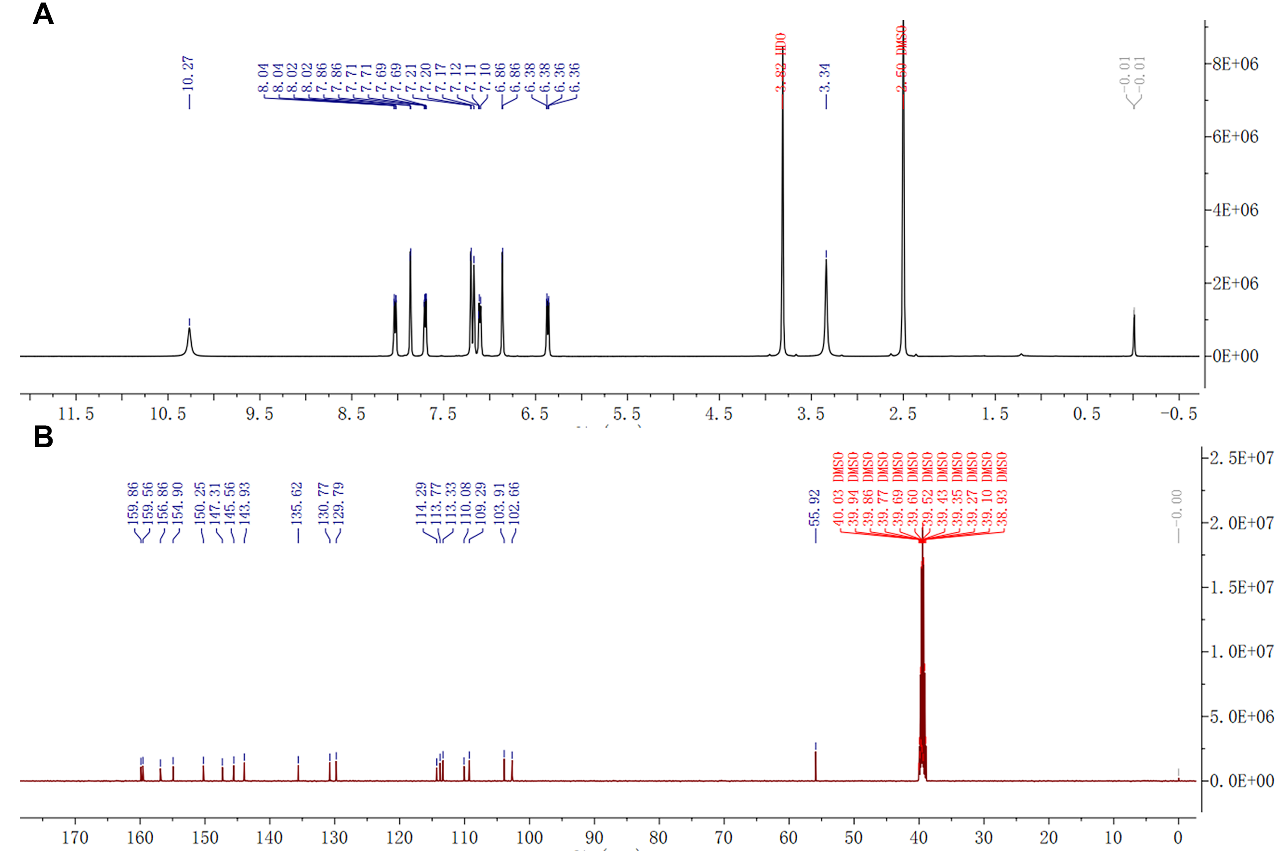
**

# Figure S3. Particle sizes of micelles loaded by different molecular weight carrier materials.


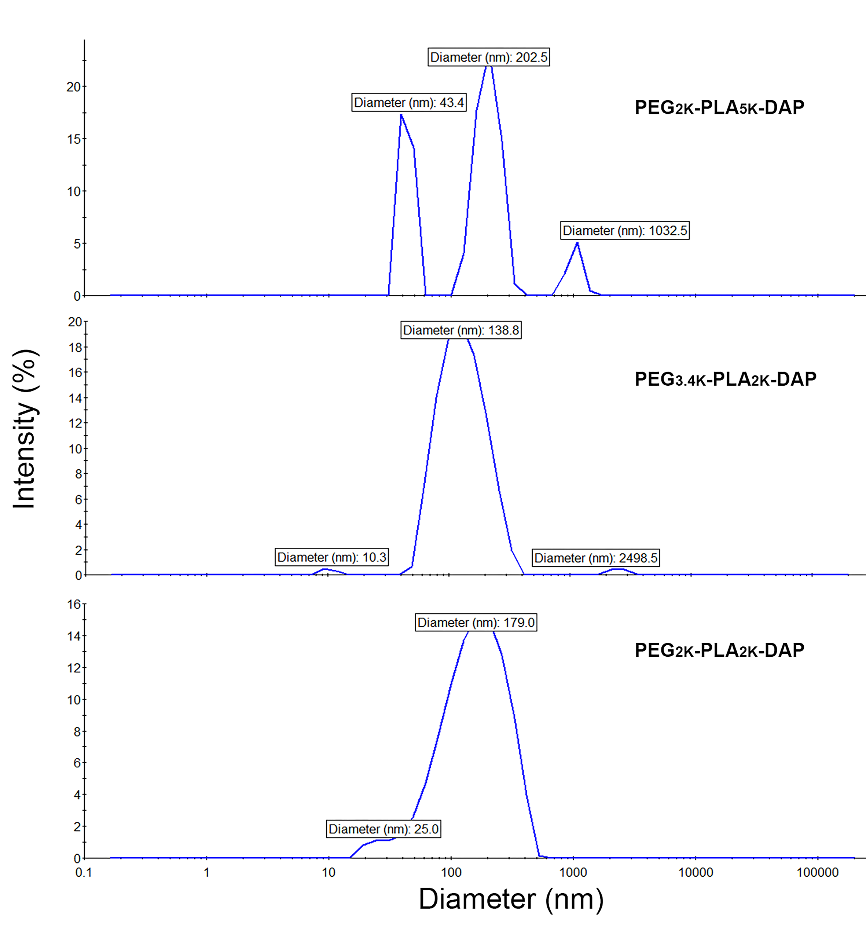


# Figure S4. Results of a univariate study. (A) Effect of drug loading ratio on the encapsulation efficiency (EE) and the drug loading (DL). (B) Effect of organic solvent type on the EE and the DL. (C) Effect of organic solvent dosage on the EE and the DL. d) Effect of hydration temperature on the EE and the DL. (E) Effect of hydration time on the EE and the DL. (F) Effect of hydration water dosage on the EE and the DL. (G) Effect of stirring time on the EE and the DL. (H) Effect of ultrasonic power on the EE and the DL.
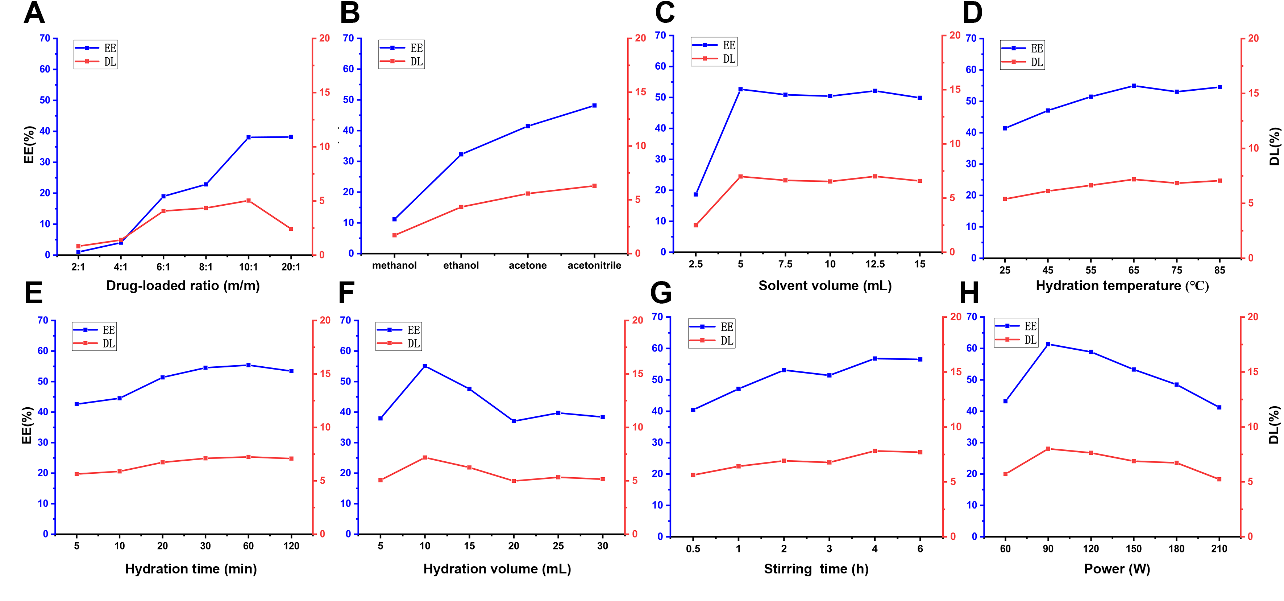


# Figure S5. Study on stability of nano-preparations in vitro. (A) Results at 0 first day. (B) Results of storing in 25℃ and 4℃ at 28th day. (C) The variation of particle size from 0 to 28 days. (D) The variation of zeta potential from 0 to 28 days.


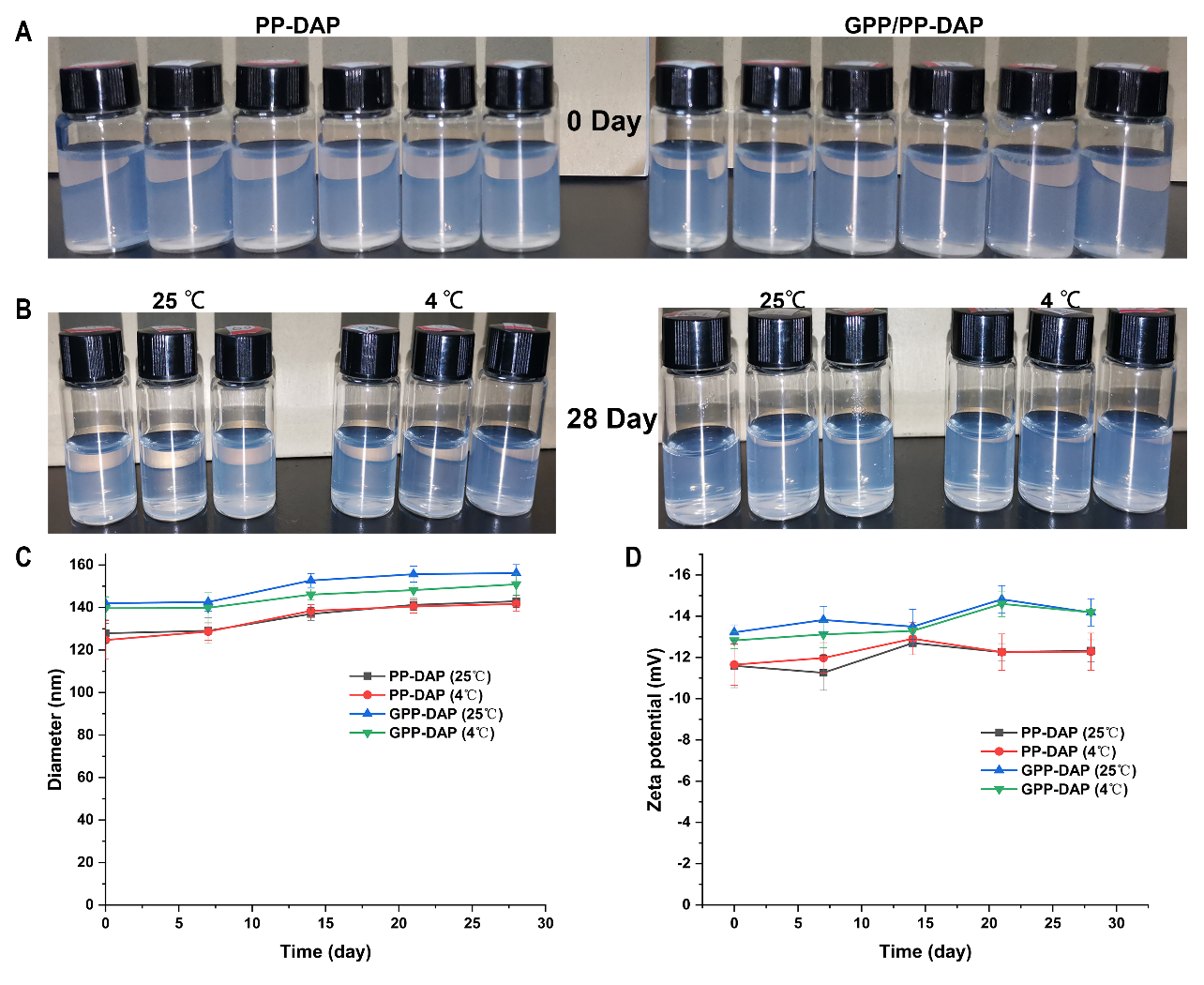


# Figure S6. Results after hemolytic assay. Positive (+); negative (−).


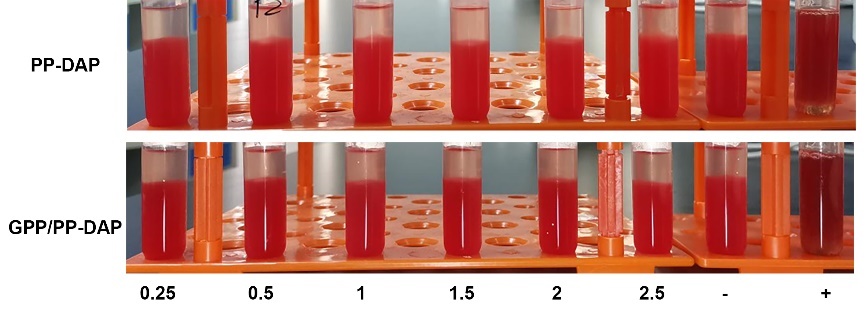


# Figure S7. The excitation and emission wavelength scanning of DAP.

#



# Figure S8. Liver lesions in mice of different days.


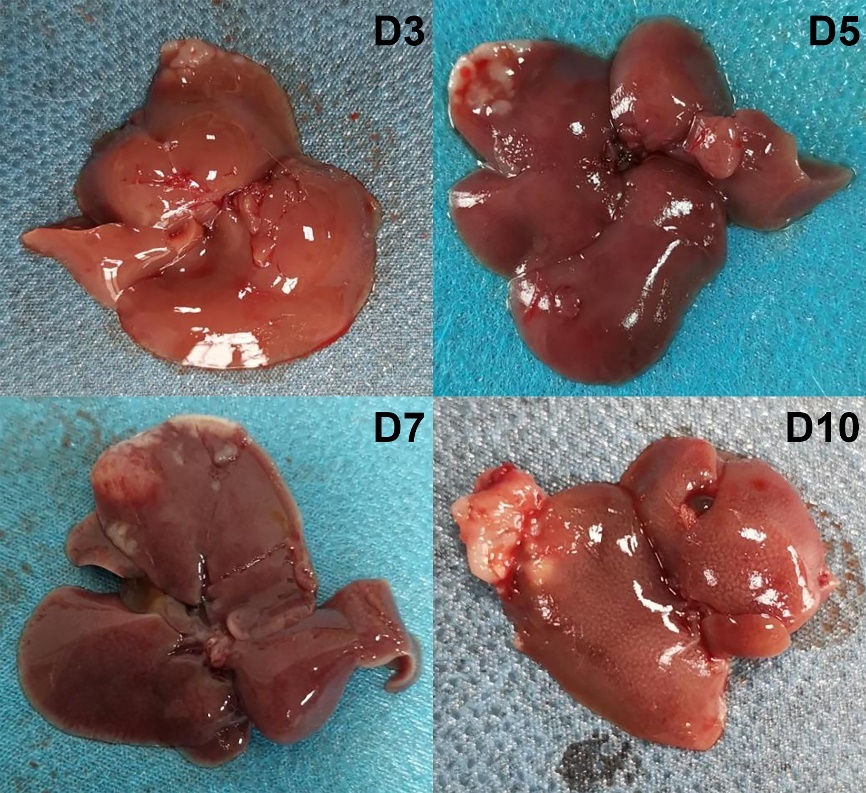


# Figure S9. The fluorescence *in vivo* imaging of DIR, PP-DIR, and GPP/PP-DIR within 48h. (A) The fluorescence distribution of mice viscera at 48h. (B) These mice were administered different DIR-tagged formulations via intraperitoneal injection at a dose of 0.5 mg/kg.


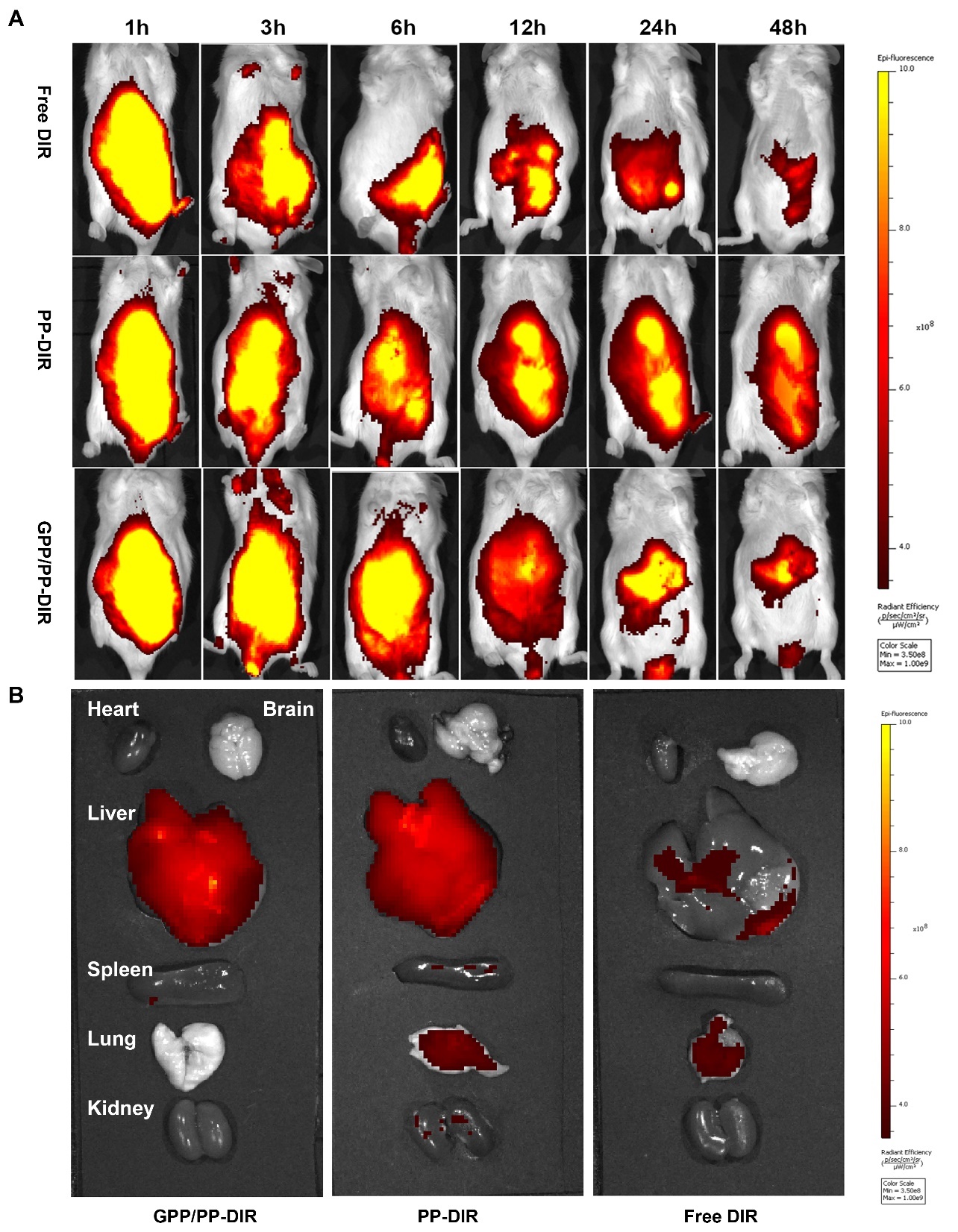

Supplement: Supplementary file 1 [file DataSheet1.docx]
